# Dual-Phase Ocular Insert with Bromfenac-Loaded PLGA MPs in a PVA Matrix for Sustained Postoperative Anti-Inflammatory Delivery

**DOI:** 10.3390/pharmaceutics17081066

**Published:** 2025-08-17

**Authors:** Farhan Alshammari, Bushra Alshammari, Asma Khalaf Alshamari, Kaushik Sarkar, Raghu Raj Singh Thakur

**Affiliations:** 1 Department of Pharmaceutics, College of Pharmacy, University of Ha’il, Hail 81442, Saudi Arabia; 2 Medical and Diagnostic Research Center, University of Ha’il, Hail 55473, Saudi Arabia; bu.alshammari@uoh.edu.sa (B.A.); ak.alshamari@uoh.edu.sa (A.K.A.); 3 Medical Surgical Nursing Department, College of Nursing, University of Ha’il, Hail 2440, Saudi Arabia; 4 Department of Chemistry, College of Science, University of Ha’il, Hail 81451, Saudi Arabia; 5 IQVIA Analytics Services Pvt. Ltd., Omega, Embassy TechSquare, Kadubeesanahalli, Bangalore 560103, India; kaushik.sarkar@iqvia.com; 6 School of Pharmacy, Queen’s University Belfast, Medical Biology Centre, 97 Lisburn Road, Belfast BT9 7BL, UK; r.thakur@qub.ac.uk

**Keywords:** ocular drug delivery system, sustained release, PLGA microparticles, bromfenac sodium, polyvinyl alcohol (PVA), postoperative ocular inflammation, dual-phase release system

## Abstract

**Background:** Postoperative ocular inflammation is a frequent complication of eye surgeries commonly managed using corticosteroids or nonsteroidal anti-inflammatory drug (NSAIDs) eye drops. However, poor ocular bioavailability and patient non-adherence due to frequent dosing limit the therapeutic efficacy of conventional eye drops. This study aimed to develop a sustained-release ocular insert containing bromfenac sodium (BS)-loaded poly(lactic-co-glycolic acid) (PLGA) microparticles (MPs) with an initial 3% (*w*/*w*) free BS fraction incorporated into a poly(vinyl alcohol) (PVA) matrix designed to achieve a dual-phase release profile for improved postoperative therapy. **Methods:** PLGA-based MPs were fabricated using a double emulsion solvent evaporation technique and incorporated into PVA films to produce ocular inserts with varying MP content. Formulations were characterized for morphology, particle size, zeta potential, drug loading, entrapment efficiency, mucoadhesion, drug distribution, and in vitro release. Data were analyzed by an ANOVA and *t*-tests with *p* < 0.05 as significance. **Results:** MPs were smooth, spherical, and well-dispersed in the PVA inserts. Particle sizes ranged from 3.7 to 5.6 µm, with drug loading 7–8% and entrapment efficiencies 47–52%. Multiphoton imaging confirmed uniform drug distribution. In vitro release showed a dual-phase profile with an initial burst followed by sustained release for up to 4 days, with only negligible further release through Day 6 in one formulation (M1-7525). **Conclusions:** The developed BS-loaded PLGA MP/PVA insert demonstrated a dual-phase release profile relevant to postoperative ocular inflammation. Its biodegradable, single-application design holds promise for enhancing compliance and therapeutic outcomes in ophthalmic care.

## 1. Introduction

Postoperative ocular inflammation is a common consequence of ophthalmic surgeries such as cataract extraction. If not properly managed, it can cause pain and sight-threatening complications such as cystoid macular edema or posterior capsule opacification. To prevent these outcomes, patients are typically prescribed intensive topical anti-inflammatory therapy and corticosteroid and/or nonsteroidal anti-inflammatory drugs (NSAIDs), following surgery [1]. However, conventional eye drops have inherent limitations. Due to rapid tear turnover and nasolacrimal drainage, less than 5% of the instilled dose is absorbed into ocular tissues [2]. Achieving and maintaining therapeutic drug levels therefore requires frequent instillation (often multiple times per day), which results in poor patient adherence. Therefore, suboptimal bioavailability and compliance with postoperative eye drop regimens contribute to inadequate inflammation control and diminished surgical outcomes [1].

Sustained-release ocular systems aim to extend drug exposure while reducing administration frequency. By prolonging drug residence time on the eye surface, such systems can improve bioavailability and reduce the burden of frequent dosing. One such approach is the dexamethasone intracanalicular insert (Dextenza^®^), which is approved by the U.S. Food and Drug Administration (FDA) and delivers anti-inflammatory therapy for up to 30 days following a single application; however, this insert is minimally invasive, requiring placement into the lower lacrimal canaliculus via punctal dilation by a healthcare professional. In some cases, manual removal through irrigation may be necessary, further emphasizing its procedural limitations [3]. This approach demonstrates the utility of sustained-release inserts and supports exploration of similar systems using alternative anti-inflammatory agents.

Bromfenac sodium (BS) is a potent ocular NSAID widely used for managing postoperative inflammation and pain [4]. It was selected for this study due to its high clinical relevance and the opportunity to enhance its delivery via sustained-release systems. BS, the salt form of bromfenac, improves aqueous solubility compared to the free acid but remains pH-dependent and is generally classified as a Biopharmaceutics Classification System (BCS) class II drug with low solubility and high permeability [5]. The presence of a bromine atom enhances its potency, providing up to four times stronger cyclooxygenase-2 (COX-2) inhibition compared to diclofenac and ketorolac, enabling effective anti-inflammatory activity at low concentrations [4]. However, BS must be formulated at an alkaline pH to achieve sufficient solubility and is prone to hydrolytic degradation in aqueous solutions during storage, making solid-state delivery an attractive approach to improve stability [6,7]. Currently, BS is marketed as a topical ophthalmic solution, including a 0.09% product (Bromday^®^) and a 0.07% formulation (Prolensa^®^), both requiring once- or twice-daily dosing for approximately two weeks post-surgery [4]. A sustained-release system could reduce dosing frequency and enhance ocular bioavailability, addressing these limitations.

Several sustained delivery approaches have been investigated, among which biodegradable polymeric microparticles (MPs) have emerged as a promising option for ocular drug delivery. In particular, poly(lactic-co-glycolic) acid (PLGA) MPs offer controlled release capabilities and biodegrade into inert byproducts, obviating the need for surgical removal. PLGA is utilized in several FDA-approved drug delivery systems and permits control over its degradation rate through variations in its copolymer composition [8]. Studies have demonstrated that PLGA MPs sustain drug release for extended periods in ocular applications; for example, PLGA MPs embedded in hydrogels provided weeks-long delivery in preclinical settings. However, the administration of PLGA MPs alone faces challenges such as burst release [9] and rapid ocular clearance [10], highlighting the importance of incorporating MPs into ocular inserts to improve retention, stability, and controlled drug delivery.

Ocular inserts offer several specific advantages in ocular drug delivery. As solid or semi-solid dosage forms placed on the ocular surface, they increase drug residence time and provide steady, controlled drug release, in contrast to conventional eye drops, which are rapidly cleared from the eye due to tear turnover, blinking, and nasolacrimal drainage [11]. By enabling accurate and reproducible dosing, reducing the required frequency of administration, and minimizing systemic drug absorption, ocular inserts enhance ocular bioavailability, improve therapeutic outcomes, and support better patient adherence to treatment regimens [12].

For the design of the ocular insert, poly(vinyl alcohol) (PVA) was selected as the matrix material due to its established safety, biocompatibility, and solubility in tear fluid, which make it suitable for ophthalmic applications. PVA has been widely used in approved ocular films and nanofilms, providing a reliable platform for drug delivery [13]. Despite advances in ocular delivery technologies, sustained-release systems for NSAIDs remain limited in postoperative care. Therefore, this study aimed to develop a novel dual-phase ocular insert composed of BS-loaded PLGA MPs embedded within a dissolvable PVA matrix, with the goal of achieving both immediate and sustained drug release. Given the high aqueous solubility of BS, the PVA matrix was designed to dissolve upon administration, providing an initial loading dose of free BS, while concurrently depositing BS-loaded PLGA MPs onto the ocular surface to sustain drug release over an extended period through a biphasic release profile.

## 2. Materials and Methods

PLGA 7502A (75:25 lactic:glycolic acid) and PLGA 5002A (50:50 lactic:glycolic acid) were sourced from Corbion Purac Biomaterials (Gorinchem, The Netherlands). PVA: molecular weight of 31,000–50,000, 98–99% hydrolysed; porcine gastric mucin; BS (≥98%, HPLC grade); and dichloromethane (≥99.8%, stabilized with amylene) were obtained from Sigma-Aldrich (Dorset, UK), and deionized water was obtained from an in-house deionization system.

### 2.1. Fabrication of MPs

MPs were prepared using the double emulsion solvent evaporation method. Specifically, 100 mg of PLGA with two different lactic-to-glycolic acid ratios (50:50 and 75:25) was dissolved in 2 mL of dichloromethane (DCM) to form the organic phase. This organic phase was then emulsified with an aqueous phase consisting of deionized water containing 10 mg of BS, using a high-speed Ultra-Turrax homogenizer (IKA, Staufen, Germany) at 14,000 rpm for 5 min to form a primary water-in-oil (W/O) emulsion. The primary emulsion was then further emulsified with 25 mL of a 1.5% *w*/*v* of PVA aqueous solution at 14,000 rpm for 5 min to produce a water-in-oil-in-water (W/O/W) double emulsion. The resulting emulsion was stirred magnetically at 250 rpm for 6 h at room temperature to allow for solvent evaporation. The MPs were centrifuged at 10,000× *g* for 15 min and washed three times with distilled water to remove unencapsulated drug. The final product was collected by vacuum filtration through filter paper and then dried under vacuum at 25 °C for 24 h.

These PLGA MPs serve as the sustained-release component of the final ocular insert; when later embedded within the dissolvable PVA matrix (Section 2.2), the combination enables a dual-phase release profile with an initial burst from the PVA phase and prolonged delivery from the loaded drug in the PLGA MPs.

### 2.2. Preparation of Ocular Inserts

To prepare the solution forming the ocular inserts, the required amount of BS was accurately weighed to achieve a concentration of 3% *w*/*w* relative to the weight of PVA in the 30% *w*/*w* aqueous solution. It was then added directly to the PVA solution and stirred using a magnetic stirrer (VWR, Dublin, Ireland) at 200 rpm at room temperature until a homogenous solution was obtained. This procedure produced a formulation consisting of 3% BS in 30% PVA. To clarify this ratio numerically, 1 mL of 30% *w*/*w* PVA solution contains approximately 0.3 g of PVA. The amount of BS corresponding to 3% *w*/*w* of this 0.3 g PVA is 9 mg. Therefore, the dry film obtained from this solution, excluding any MPs, contains 300 mg of PVA and 9 mg of BS, giving a BS-to-PVA ratio of 3%. Varying amounts of BS-loaded MPs were then added to this solution while stirring to produce the different formulations described in Table 1.

In formulations containing MPs, 1% or 2% *w*/*w* of BS-loaded PLGA MPs (relative to the PVA weight) were added, equivalent to 3 mg or 6 mg per mL of solution, respectively. The cumulative drug release was calculated based on the total theoretical BS content per insert, incorporating both the 3% free BS and the additional BS encapsulated within the MPs, as determined by the encapsulation efficiency reported in Section 2.1.

**Figure 1:**
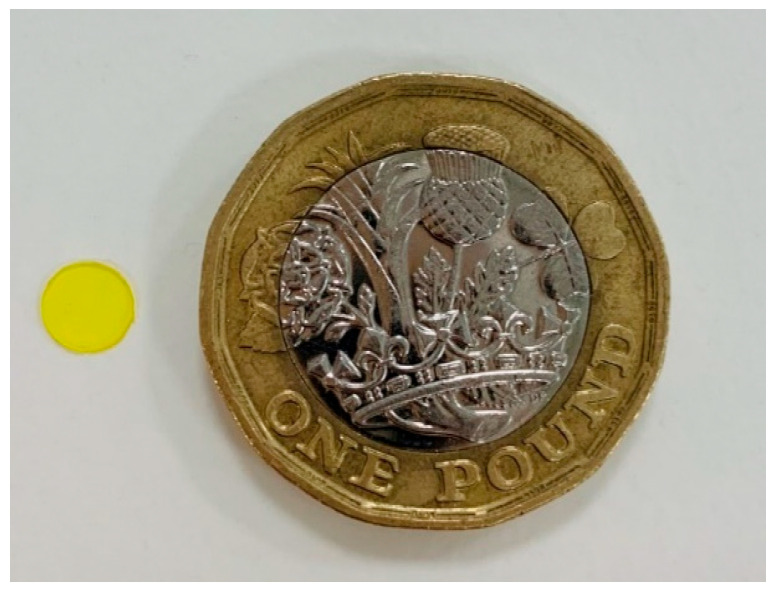
Ocular insert placed next to a British one-pound coin for scale.

Each formulation was fabricated by casting 1 mL of the resulting mixture into plastic weighing boats (3 cm × 3 cm), which were then left to dry at 25 °C for 24 h to form thin films. After complete drying, the films were carefully peeled off and cut into round inserts using a single-hole puncher with a 6 mm diameter, as shown in Figure 1. PVA-only inserts were visually transparent, while microparticle-loaded inserts showed slightly reduced transparency due to the dispersed small PLGA MPs. All inserts were immediately wrapped in non-stick wax paper and stored in a vacuum desiccator at 25 °C to prevent moisture absorption until further analysis.

In this system, the water-soluble PVA matrix provides the immediate-release phase by rapidly dissolving in tear fluid and releasing the dispersed BS as an initial burst. In contrast, BS encapsulated within PLGA MPs forms the sustained-release phase, with drug diffusion and polymer erosion extending delivery over several days.

### 2.3. Characterization of BS-Loaded MPs and Ocular Inserts

The fabricated inserts and MPs underwent comprehensive evaluations to assess their morphological characteristics, mucoadhesive properties, and drug distribution.

**Morphological Analysis:** The morphology of PLGA-based MPs prepared with PLGA 75:25 and PLGA 50:50 was examined using a Hitachi TM 3030 Tabletop SEM (Hitachi High-Technologies Europe GmbH, Krefeld, Germany). Samples were mounted on aluminum stubs using carbon tape and sputter-coated with gold to enhance conductivity. Imaging was conducted at an appropriate acceleration voltage, and micrographs were captured for analysis. Additionally, SEM imaging was used to evaluate the surface morphology of both MP-loaded and blank (drug-free) inserts, allowing comparison of structural integration and uniformity within the polymeric matrix.

**Particle Size Analysis:** The particle size distribution of the MPs was assessed using laser diffraction (Sympatec HELOS, Clausthal-Zellerfeld, Germany) equipped with a specialized glass cuvette for small-volume samples. An AR2 lens (measuring range: 0.25–877 µm) was utilized during the analysis. Samples were introduced into the dispersion unit and stirred at 2000 rpm until an obscuration level between 20% and 40% was achieved, ensuring optimal measurement conditions. The key particle size parameter determined is X90, which indicates that 90% of the particles fall below the specified size. Each sample was measured in 3 replicates to ensure reproducibility. In addition to these parameters, the surface mean diameter (SMD) and volume mean diameter (VMD) were also calculated to provide further insight into the average particle size based on surface area and volume distribution

**Zeta potential:** The surface charge of the MPs, which is critical for their colloidal stability and potential interaction with biological tissues, was evaluated through zeta potential analysis. MPs were dispersed in deionized water at a low concentration (1 mg/mL), and 1 mL of the suspension was transferred into a Folded Capillary Zeta Cell (Malvern Instruments Ltd., Malvern, UK). The zeta potential was measured using a Zetasizer (Malvern Instruments Ltd., Malvern, UK), based on the electrophoretic mobility of charged particles under an applied electric field.

**Drug content analysis:** To quantify the drug content within the MPs, 10 mg of each type of MP (PLGA 75:25 and 50:50) was dissolved in 5 mL of 0.1 M sodium hydroxide (NaOH) and agitated in a horizontal shaker for 12 h to ensure complete hydrolysis of the PLGA polymer. The resulting clear solution was centrifuged at 1726× *g* for 15 min to remove any undissolved residues. A calibration curve was prepared using standard solutions of BS in 0.1 M NaOH at concentrations of 0.15625, 5, 10, 25, 50, and 100 µg/mL, with 0.1 M NaOH serving as the blank. Absorbance was measured at 270 nm using a microplate reader (BMG LABTECH, Offenburg, Germany) to establish the linear relationship between absorbance and concentration. The supernatant from the dissolved samples was analyzed in triplicate under the same conditions. The polymer matrix did not interfere at the selected wavelength, ensuring accurate quantification of BS. Drug content analysis was performed for MPs only, as their fabrication process involves steps that may lead to drug loss, unlike the insert preparation, which involves simple mixing and casting without further loss.

Based on the measured drug content, the following calculations were performed:

$$Drug\;loading\;\left(\%\right)=\frac{weight\;of\;drug\;in\;MPs}{weight\;of\;MPs}\times 100$$

$$Entrapment\;efficiency\;\left(\%\right)\;=\frac{weight\;of\;drug\;in\;MPs}{weight\;of\;drug\;fed\;initially}\times 100$$

**Figure 2:**
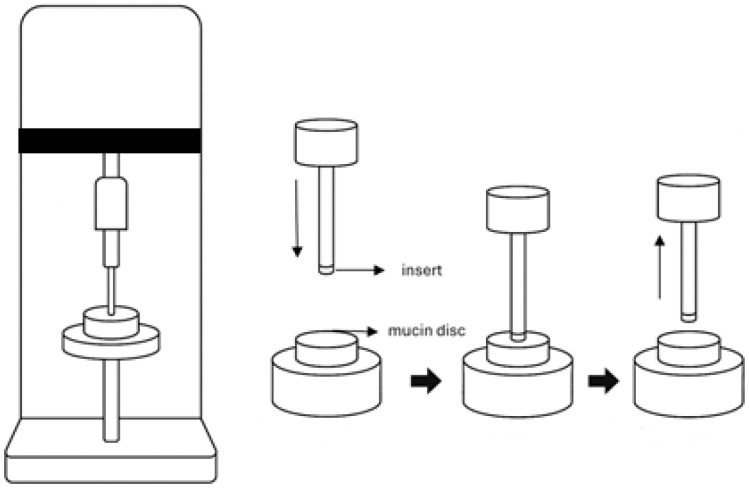
Schematic diagram illustrating the experimental setup for the mucoadhesion test using a texture analyzer. Redrawn by the authors based on the method described in Cegielska et al., 2022 [14].

**Mucoadhesive Properties:** The mucoadhesive strength of the ocular inserts was evaluated using a TA.XT2 Texture Analyser (Stable Micro Systems, Surrey, UK) (Figure 2). Mucin discs (400 mg) were prepared by compressing porcine gastric mucin using a 13 mm evacuable pellet press at a force of 10 tonnes for one minute. This setup simulated the ocular mucosal surface for in vitro adhesion testing. The force required to detach each insert from the mucin disc was measured and recorded as the bioadhesive strength, reflecting the formulation’s potential to adhere to the ocular surface and prolong residence time. Each insert was mounted onto a polycarbonate probe using double-sided adhesive tape. Prior to testing, mucin discs were pre-wetted with a 5% mucin solution, and any excess fluid was gently removed by blotting. The probe was lowered at a speed of 1 mm/s until a contact force of 0.1 N was achieved between the insert and the mucin disc. After maintaining contact for 10 s, the probe was withdrawn at the same speed (1 mm/s), and the detachment force was recorded.

**Drug Distribution Analysis:** Multiphoton microscopy was employed to visualize the distribution of BS within the MP-loaded insert (M1-5050). Imaging was conducted using a Leica SP5 multiphoton microscope (Leica Microsystems, Wetzlar, Germany) equipped with a titanium-sapphire laser. Imaging was performed at a laser excitation wavelength of 820 nm, with emission collected between 400 and 450 nm. The resulting micrographs were analyzed to evaluate the uniformity of drug distribution within the insert structure.

**In Vitro Drug Release:** In vitro release studies were performed by placing a single ocular insert in 1 mL of phosphate-buffered saline (PBS, pH 7.4) in 1 mL Eppendorf tubes [15]. The tubes were incubated at 37 °C in a shaking incubator at 90 cycles/min. This agitation rate was chosen to mimic tear fluid movement and maintain sink conditions while avoiding excessive shear, consistent with reported sustained ophthalmic release methods [16]. At predetermined time intervals, the entire 1 mL sample was centrifuged at 2185× *g* for 5 min, and 100 µL of the clear supernatant was withdrawn and analyzed by UV-Vis spectrophotometry at 270 nm. Centrifugation before sampling was used to remove suspended PLGA MPs and prevent UV absorbance interference. Withdrawn volumes were replaced with fresh PBS to reduce the potential for medium saturation. The selected volume was considered adequate to ensure measurable and consistent drug release under the study conditions. Drug concentrations were calculated using a validated calibration curve (0.15625–250 µg/mL), with confirmed linearity, accuracy, precision, and specificity.

The cumulative drug release (%) at each time point was calculated relative to the total theoretical drug content in each ocular insert. This total included both the BS incorporated directly at 3% *w*/*w* relative to PVA and the additional BS encapsulated within the PLGA MPs. The amount of encapsulated BS was determined based on the known encapsulation efficiency measured during microparticle characterization. This combined value was used as the 100% reference for plotting the drug release profiles. All experiments were performed in triplicate.

**Statistical Analysis:** Statistical analysis of the mucoadhesive strength of different inserts and BS release from various formulations was performed using a two-way analysis of variance (ANOVA). In addition, particle size analysis, zeta potential, and drug content data were analyzed using unpaired *t*-tests. A *p*-value of <0.05 was considered statistically significant. All statistical analyses were conducted using GraphPad Prism Version 5 (GraphPad Software Inc., San Diego, CA, USA).

## 3. Results and Discussion

### 3.1. Morphological Analysis

**Figure 3:**
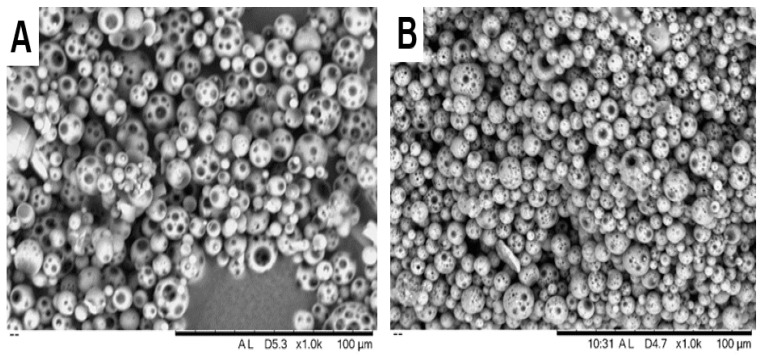
SEM images of BS-loaded PLGA MPs. Representative images show (**A**) M1-5050 and (**B**) M1-7525 formulations, both exhibiting uniformly spherical morphology with smooth surfaces.

SEM imaging confirmed that the BS-loaded PLGA MPs produced by double emulsion were uniformly spherical and smooth (Figure 3), which is suggestive of efficient drug encapsulation within the MP matrix. Both PLGA 50:50 and 75:25 MPs exhibited comparable morphology, characterized by well-separated individual particles and sub-10 µm spheres with no obvious differences due to polymer composition. This morphology is consistent with effective emulsification and polymer hardening and suggests that altering the lactic:glycolic ratio did not adversely affect particle formation.

The visual attributes of the inserts are important for patient comfort and clinical usability. The PVA-only inserts (without MPs) were visually transparent due to the inherent clarity of the PVA matrix, consistent with previous reports [17]. In contrast, the MP-loaded inserts exhibited slightly reduced transparency because of the dispersed PLGA MPs; however, the inserts still appeared sufficiently clear for ocular application, and the drug was dissolved in the matrix without visible crystallinity. Given their small size (6 mm diameter), relatively small particle size (<10 µm), and placement in the lower conjunctival sac away from the visual axis, the inserts are not expected to interfere with vision. Formal light transmittance or transparency testing was not performed in this study and should be addressed in future work.

**Figure 4:**
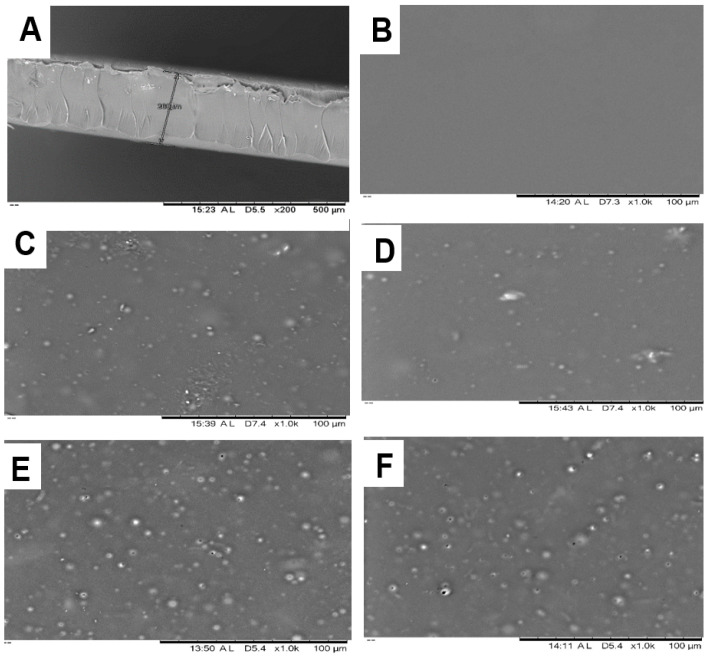
(**A**) Cross-sectional SEM image of M0 insert thickness (260 μm) and SEM images of surfaces of (**B**) blank insert (M0) and (**C**) M1-5050, (**D**) M1-7525, (**E**) M2-5050, (**F**) M2-7525 inserts.

The PVA insert morphology by SEM revealed successful integration of MPs into the insert. Blank inserts (M0) had a homogenous, smooth surface, whereas MP-loaded inserts (M1-5050, M1-7525, M2-5050, and M2-7525) showed spherical MP protrusions uniformly distributed across the matrix (Figure 4). The MPs remained well-dispersed even at 2% loading, indicating no aggregation and homogenous composition during casting. Cross-sectional images of the insert (~260 µm thickness) confirmed MPs embedded throughout the PVA layer without voids or cracks. This intact structure demonstrates that incorporating up to 2% (*w*/*w*) of PLGA MPs did not compromise the insert’s mechanical integrity. Notably, Mirzaeei et al. [18] reported uniform nanoparticle dispersion within PVA-based inserts, yielding smooth, defect-free films with acceptable mechanical strength. However, their formulation involved a complex matrix of multiple polymers. In contrast, our study demonstrates a simplified dual-phase system in which BS-loaded PLGA MPs are uniformly embedded within a single-component PVA matrix. This design maintains structural integrity while enabling biphasic drug release, characterized by an initial burst phase followed by sustained delivery, without requiring additional mucoadhesive agents. The streamlined composition supports reproducibility, scalability, and clinical applicability, offering a potentially superior alternative for postoperative ocular therapy.

### 3.2. Particle Size Analysis and Zeta Potential

Laser diffraction showed a narrow, monomodal size distribution for both formulations (Table 2). PLGA 50:50 MPs had a larger volume–mean diameter (~5.6 µm) compared to PLGA 75:25 MPs (~3.7 µm), a statistically significant difference (*p* < 0.05). This trend likely arises from differences in intrinsic polymer properties, as the 50:50 copolymer (with higher glycolic acid content) tends to produce slightly larger droplets during emulsification, whereas the 75:25 copolymer (with higher lactic acid content) yields smaller particles under the same conditions [19]. The droplet size during emulsification, and consequently the MPs’ size, is also strongly influenced by interfacial phenomena and the chemical nature of the materials used. PVA, acting as an emulsifier, lowers the interfacial tension between the PLGA organic phase and the aqueous continuous phase. An adequate PVA concentration stabilizes the interface, preventing coalescence and promoting the formation of smaller droplets, whereas insufficient interfacial stabilization results in larger particles [20].

It is well-established that the composition of PLGA influences both MP formation and degradation kinetics. Polymers with a higher glycolic acid content, such as PLGA 50:50, are more hydrophilic, tend to form larger droplets during emulsification, and degrade more rapidly. In contrast, PLGA with a higher lactic acid content, such as the 75:25 ratio, is more hydrophobic, typically forming smaller particles that exhibit slower degradation rates [21]. The results are consistent with this observation. Although the 75:25 MPs exhibited smaller particle sizes, which would typically favor faster release due to higher surface area, the inherently slower hydrolysis rate of the lactide-rich PLGA 75:25 copolymer is known to result in slower release [22].

Both types of MPs exhibited a moderately negative zeta potential, ranging from approximately −23 to −25 mV, with no statistically significant difference between the formulations. This negative surface charge is characteristic of PLGA particles, resulting from carboxyl end-groups, and contributes to colloidal stability through electrostatic repulsion. Notably, the comparable zeta potentials between the 50:50 and 75:25 formulations suggest that polymer chemistry did not substantially affect the surface charge density. Therefore, the observed differences in formulation performance are more likely attributable to variations in particle size and polymer degradation behavior, rather than differences in colloidal stability.

### 3.3. Drug Loading and Encapsulation Efficiency

Both PLGA MPs formulations achieved high BS loading, approximately 7–8% *w*/*w*, with encapsulation efficiencies ranging from 47% to 52% (Table 3), and no statistically significant differences were observed between the 50:50 and 75:25 compositions (*p* > 0.05). This suggests the double-emulsion method was reproducible and effective for BS, yielding consistent entrapment in both polymer types. Achieving about 51% entrapment of the highly water-soluble BS is notable as PLGA MPs often suffer some drug loss to the external phase during fabrication. The encapsulation efficiency achieved for BS ranged from 46% to 51%, which is consistent with values reported for hydrophilic drugs encapsulated in PLGA-based systems, where drug loss to the external aqueous phase during fabrication is a well-recognized challenge. This level of entrapment for a water-soluble drug such as BS highlights the effectiveness and reproducibility of the double-emulsion technique [21].

Efficient drug encapsulation is essential for sustained-release delivery systems, as it ensures that the majority of the BS resides within the MPs rather than on the surface, thereby minimizing initial drug loss. A slightly higher mean drug loading was observed in the 75:25 PLGA MPs (7.91%) compared to the 50:50 formulation (6.93%); however, this variation was small and within experimental error and therefore may not clearly relate to formulation aspects. However, this difference did not result in a significant variation in the release profile attributable to drug loading. Overall, both formulations exhibited consistent and efficient drug incorporation, providing a reliable reservoir of BS within the MPs to support the sustained-release phase.

### 3.4. Mucoadhesive Properties

**Figure 5:**
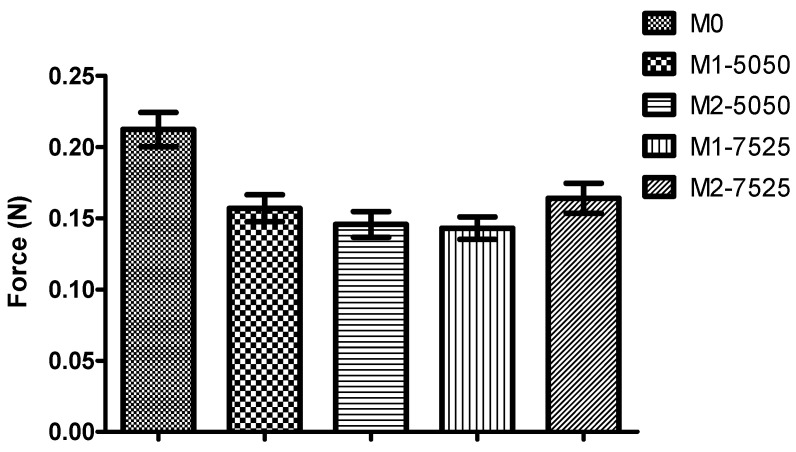
Mucoadhesive strengths of different formulations of ocular inserts. (Mean ± SD, *n* = 3).

All insert formulations exhibited measurable mucoadhesive strength in a texture analysis, indicating their potential to adhere to the ocular mucin layer (Figure 5). Interestingly, the plain PVA film (M0) demonstrated the highest detachment force, which is about 0.21 newton (N), while the incorporation of MPs in the formulations significantly reduced mucoadhesion (*p* < 0.0001 vs. M0). Inserts containing 2% MP loading exhibited comparable adhesion to those with 1%, with no statistically significant differences between them. The lowest mucoadhesive force, which is about 0.14 N, was observed for M1-7525.

This finding contradicts the mechanical theory of mucoadhesion, which proposes that surface roughness introduced by PLGA MPs creates additional anchor points that enhance adhesion by increasing the interfacial area and facilitating interlocking with the mucin network [23]. However, the opposite effect was observed. One plausible explanation is that the presence of MPs disrupted the uniformity and cohesive integrity of the PVA film, potentially reducing the effective contact area available for mucin interaction. Additionally, the MPs may have interfered with intermolecular hydrogen bonding between PVA and mucin glycoproteins [24], which is critical for mucoadhesion. Instead of facilitating interlocking, the discrete MPs may have acted as non-interactive discontinuities, weakening the cohesive forces across the interface. Moreover, depending on their distribution and surface chemistry, MPs may reduce the wettability or flexibility of the film surface, both of which are essential for forming intimate contact with the mucous layer.

### 3.5. Drug Distribution Analysis

**Figure 6:**
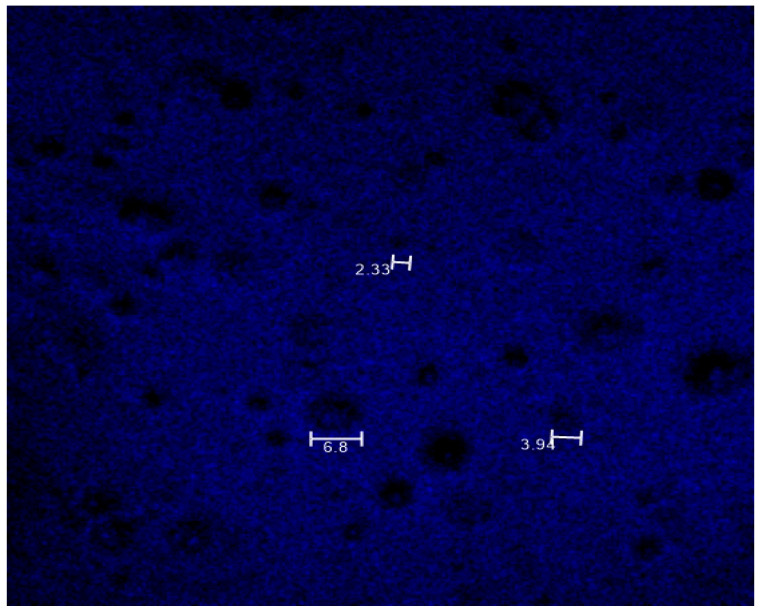
Multiphoton imaging of BS loaded within MP-loaded insert (M1-5050) at laser excitation of 820 nm and emission collected between 400 and 450 nm. Numerical annotations in the image show representative MP diameters in µm.

Figure 6 presents the multiphoton fluorescence image of MP-loaded PVA inserts. A diffuse blue signal is observed across the insert, indicating widespread distribution of BS within the polymer matrix. Distinct circular regions, approximately 2 to 7 µm in diameter, are visible and correspond to embedded PLGA MPs. These regions exhibit slightly lower fluorescence intensity than the surrounding PVA matrix, suggesting partial localization of BS within the MPs’ cores. This reduced intensity may result from lower local drug concentrations or fluorescence quenching within the polymer environment.

Such intensity gradients are consistent with interpretations reported in multiphoton imaging studies, where signal variations within polymer-based drug delivery systems have been attributed to heterogeneous drug distribution, differences in encapsulation efficiency, or local quenching effects inside carrier structures. Specifically, prior work has shown that fluorescence intensity does not always scale linearly with dye loading, as high local concentrations and the physicochemical characteristics of the polymeric core may suppress signal expression [25]. Together, these observations support the biphasic design of the formulation, in which the PVA matrix contains freely dispersed BS for immediate release, while the PLGA MPs serve as reservoirs for sustained delivery. The absence of large drug-free voids or highly concentrated aggregates further supports a reasonably uniform drug distribution across the insert.

### 3.6. In Vitro Drug Release

**Figure 7:**
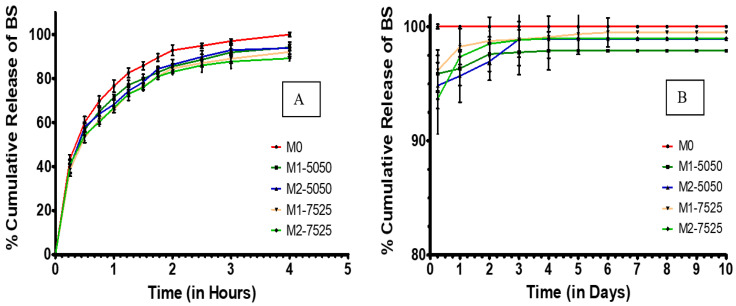
Cumulative BS release (**A**) in hours and (**B**) in days from different formulations of ocular inserts. (Mean ± SD, *n* = 3).

The in vitro release profiles revealed generally similar biphasic patterns across the insert formulations, with some variation in release rates and extent. Notably, M0 inserts (which contained no MPs) demonstrated a rapid-release profile, releasing approximately 80% of BS within one hour and achieving nearly 100% release by the fourth hour. This pattern is indicative of a fast-release profile of hydrophilic drugs, similar to diffusion-based films, and confirms the lack of sustained-release behavior. In contrast, all inserts containing MPs (in M1-5050, M1-7525, M2-5050, and M2-7525 formulations) exhibited an initial burst release followed by a markedly delayed release, taking several days to reach nearly 100% cumulative release. This distinct delay highlights the impact of the PLGA MPs on sustaining the drug release (Figure 7). This dual-phase pattern was intentionally designed to meet therapeutic needs by combining a loading dose for immediate drug levels with prolonged exposure from an encapsulated drug. The initial burst phase, a 60–80% release within the first hour, resulted from 3% free BS deliberately incorporated into the PVA matrix to ensure rapid availability. The remaining drug content was encapsulated in PLGA MPs, which gradually released BS. Since unencapsulated BS was removed during MP purification by centrifugation and washing, the observed burst effect cannot be attributed to residual surface drugs. Instead, it is a planned component of the dual-phase delivery strategy.

During the first 30 to 60 min, M1-5050, M1-7525, M2-5050, and M2-7525 released approximately 60 to 80% of the drug. Following this burst phase, the inserts continued to release BS in a controlled and sustained manner over several subsequent days. In the initial hours, (M1-7525 and M2-7525) exhibited a slightly slower burst release compared to (M1-5050 and M2-5050), which may reflect the more hydrophobic nature of PLGA 75:25 limiting immediate water ingress and slowing the drug release [22]. The corresponding drug release rate profiles (µg/day) for each formulation are shown in the Supplementary Materials (Figure S1). Between Day 1 and Day 3, however, (M1-7525 and M2-7525) released BS more rapidly, surpassing (M1-5050 and M2-5050). This shift suggests that, once hydrated, the smaller particle size of the 75:25 MPs facilitated faster diffusion due to a larger surface-area-to-volume ratio [26].

From 4 h to 7 days (Figure 7B), the curves flattened into a sustained-release phase governed by the BS slowly diffusing out of the PLGA MPs or by the PLGA MPs degrading. By Day 1, both the 50:50 and 75:25 PLGA MP-loaded inserts exhibited comparable cumulative release profiles, with the 50:50 formulation showing a slightly higher release percentage. The difference, however, was modest and became more pronounced over the following days. By Day 4, both 50:50 and 75:25 PLGA MP-loaded inserts exhibited nearly complete drug release, approaching 100% cumulative release. While the 50:50 formulations showed a slightly faster release rate in the early stages, the difference between the two polymer types diminished by Day 6. These findings suggest that under the current formulation and testing conditions, PLGA composition had only a minor influence on long-term release kinetics, despite the expected slower degradation of the more hydrophobic 75:25 polymer [21].

Statistical comparisons confirmed the advantage of incorporating MPs. All MP-loaded inserts released significantly less of the drug in the early hours and released the drug more gradually over subsequent days compared to the burst-only blank film (*p* < 0.0001). Although a statistically significant difference between the 50:50 and 75:25 formulations was observed at 24 h and on Day 7 (*p* < 0.05), this difference was modest and did not translate into a substantial divergence in overall release behavior. This suggests that while polymer composition can influence release kinetics, its effect was limited under the formulation conditions used (PLGA/PVA ratio, 3% drug loading, and defined emulsification parameters).

The release profiles revealed only modest differences in drug release performance between the 50:50 and 75:25 PLGA-based inserts. Although the 50:50 formulations exhibited a slightly faster release during the first 24 to 48 h, all MP-loaded inserts, regardless of polymer composition, achieved nearly complete release, approximately 98 to 100 percent, by Day 6. These findings indicate that under the current formulation parameters and drug load, the effect of PLGA composition on overall release duration was limited. Nonetheless, the platform successfully achieved biphasic delivery characterized by an initial burst followed by sustained release.

Modest modulation of the release rate through polymer selection remains relevant, particularly for future applications requiring extended release durations or higher drug payloads. Another contributing factor to the sustained release behavior is the gradual dissolution of the PVA matrix, which progressively exposes embedded PLGA MPs and supports prolonged drug delivery, as demonstrated in similar systems where PLGA microspheres embedded in PVA hydrogels showed extended burst phases due to diffusional resistance caused by the hydrogel barrier [27].

In evaluating the clinical relevance of the observed release profiles, it is important to consider the typical therapeutic dosing requirements of BS in ophthalmic applications. Commercial BS eye drops are usually formulated at 0.09% *w*/*v*, delivering approximately 45 μg per drop [28]. With a standard twice-daily dosing regimen and assuming an ocular bioavailability of approximately 5%, the effective therapeutic dose reaching ocular tissues is estimated at 4.5 μg per day. In the present study, MP-loaded inserts exhibited a biphasic release pattern, with an initial burst phase followed by a sustained release lasting approximately 4 days, with the formulation (M1-7525) showing only minimal additional release through Day 6. The sustained-release phase accounted for approximately 15–20% of the total drug load, as observed in Figure 7B. Based on the total drug content incorporated into each insert, this corresponds to an estimated average daily release rate that closely approximates the 4.5 μg/day therapeutic target. This alignment suggests that the developed ocular inserts may offer a promising alternative to conventional eye drops, with potential to provide effective postoperative inflammation management and reduced dosing frequency.

This delivery duration is comparable to that of the well-established Ocusert^®^ insert, which provides a 7-day release, and is competitive with other sustained ocular drug delivery systems such as intracanalicular dexamethasone inserts, which extend to 30 days but require clinical placement. Of particular clinical interest is the dual-phase profile of the current system, which provides a high initial dose followed by sustained drug release. This pattern is especially advantageous for managing postoperative inflammation, where immediate therapeutic levels are needed to control acute symptoms, followed by maintenance dosing to support recovery and reduce the need for repeated administration [29].

No significant difference was observed between the 1% and 2% MP-loaded inserts within the same polymer type (*p* > 0.05), indicating that doubling the MP content did not significantly extend the release duration. It is likely that even the 1% MP inserts contained sufficient particles to support multi-day release. The additional MPs in the 2% inserts contributed only slightly to the sustained phase, while the rate-limiting step remained governed by the degradation characteristics of the PLGA polymer rather than the MP concentration.

The initial burst release serves as a loading dose to rapidly achieve therapeutic levels for managing acute inflammation or pain, while the subsequent sustained phase maintains drug levels without frequent re-dosing. This biphasic strategy, supported by prior studies for wound healing and infection control, is particularly relevant for NSAIDs, where early COX-2 inhibition curbs inflammatory cascades and sustained release prevents rebound inflammation. In our inserts, maintaining a constant free drug fraction ensured comparable initial bursts in M1-5050, M1-7525, M2-5050, and M2-7525, while the sustained phase varied slightly with MP content. This pattern is consistent with findings by Eck et al. who reported that short-term release is governed by the amount of a drug in the polymer matrix, whereas long-term delivery is controlled by the particulate fraction [30].

## 4. Implications

The dual-phase ocular insert developed in this study meets its intended objectives by providing an initial burst release of BS that mimics a loading dose, followed by sustained release for approximately 4 days, with negligible further release through Day 6 in one formulation (M1-7525). This design addresses the limitations of conventional eye drops by maintaining therapeutic drug levels with a single application, thereby enhancing patient adherence.

BS is a potent NSAID that typically requires once- or twice-daily administration in eye drop form. The developed insert, by contrast, could potentially replace multiple instillations with a single post-surgical application. Although the current findings are based on in vitro studies, the release profile supports further evaluation, including progression to ex vivo testing to better simulate physiological conditions. Maintaining BS delivery for about 4 days, with negligible further release to Day 6 in one formulation (M1-7525), may offer consistent anti-inflammatory action during the critical postoperative period. An additional future step could involve evaluating a control insert containing only encapsulated drug (without free BS) to further isolate the role of PLGA MPs in sustained release.

Zhang et al. [31] demonstrated that sustained BS delivery from a PLGA-based intraocular implant effectively reduced inflammation and posterior capsule opacification in a rabbit cataract model. While our insert differs by being topically applied and fully dissolvable, both systems rely on sustained NSAID release to support improved surgical outcomes.

This insert offers a minimally invasive alternative composed of soluble PVA and biodegradable PLGA MPs that gradually erode, eliminating the need for surgical removal. This feature is comparable to other biodegradable platforms such as the dexamethasone intracanalicular plug, with the added benefit of potential self-administration. The performance of our insert compares favorably with established systems. For example, Ocusert achieved a seven-day zero-order release of pilocarpine, and our formulation similarly provides extended release of a modern NSAID using a simple matrix–particle system [32].

Although these results are based on in vitro conditions and physiological factors such as tear turnover and blinking may alter drug release and retention, the observed mucoadhesive properties suggest that the inserts could maintain sufficient residence time on the ocular surface. This prolonged contact may help sustain drug levels and reduce the need for frequent dosing. However, further studies using ex vivo ocular models or appropriate in vivo animal systems are necessary to evaluate pharmacokinetics and therapeutic effectiveness under more biologically relevant conditions.

In summary, the BS-loaded PLGA MP/PVA insert integrates both immediate and sustained drug release within a biodegradable platform. While the current findings are encouraging, they remain limited to in vitro experiments. The demonstrated release characteristics and insert performance support the need for continued investigation, particularly through ex vivo and subsequent in vivo studies, to assess the system’s clinical translation potential and to investigate the long-term stability of the inserts, including their drug potency retention and release profile under storage conditions, as part of further development [33].

## 5. Conclusions

This study reports the development and in vitro evaluation of a dual-phase ocular insert designed to improve drug delivery in the postoperative setting. By incorporating BS-loaded PLGA MPs into a dissolvable PVA matrix, the system enables an initial burst release for an early therapeutic effect, followed by sustained drug delivery for up to 4 days, with further release through Day 6 in one formulation (M1-7525). This biphasic release profile aligns with the therapeutic demands of postoperative inflammation management, which often requires both immediate and sustained drug availability.

Characterization showed uniform microparticle morphology, efficient drug loading, and successful integration of MPs within the polymer matrix without compromising film integrity. Although the inclusion of MPs resulted in reduced mucoadhesive strength, all tested formulations maintained acceptable bioadhesion for ocular application. The drug release profiles demonstrated nearly complete BS release by Day 4, with minimal extension to Day 6 observed only in one formulation (M1-7525), with minor variations based on PLGA composition.

The resulting insert offers a biodegradable, dissolvable alternative to conventional NSAID eye drops and to sustained-release systems that require clinical removal. By reducing the need for frequent instillations, this delivery platform may facilitate improved dosing convenience and therapeutic consistency. Future ex vivo and in vivo studies are warranted to further evaluate the insert’s pharmacokinetic behavior, retention time, and clinical efficacy, and to assess the long-term stability of the inserts to confirm drug content retention and consistent release behavior under defined storage conditions.

## Figures and Tables

**Table 1:** Composition of ocular inserts: the type of PLGA used in BS-loaded MPs and the amount of MPs added relative to the weight of the PVA solution used for insert fabrication.

Formulation Code	Type of PLGA in MPs (Lactic: Glycolic Ratio)	Amount of MPs (% *w*/*w*) in the Inserts
M0	N/A	0
M1-5050	50:50	1
M1-7525	75:25	1
M2-5050	50:50	2
M2-7525	75:25	2

**Note:** All formulations contained a fixed concentration of 30% *w*/*v* PVA solution with 3% *w*/*w* BS. The table specifies both the type of PLGA used in MPs (lactic:glycolic acid ratio) and the amount of MPs incorporated into the inserts, as prepared in Section 2.1.

**Table 2:** Particle size analysis of PLGA MPs (SMD, VMD, and X_90_) prepared using PLGA 50:50 and 75:25 polymers by laser diffraction. Data are presented as mean ± standard deviation (SD) (*n* = 3).

Type of MPs	SMD (µm) ± SD	VMD (µm) ± SD	X90 (µm) ± SD
PLGA 50:50-based MPs	3.52 ± 0.61	5.57 ± 1.10	5.54 ± 1.02
PLGA 75:25-based MPs	2.55 ± 0.32	3.70 ± 0.44	3.48 ± 0.53

**Table 3:** Drug loading and entrapment efficiency of BS in PLGA MPs prepared with two different lactic:glycolic acid ratios. Data are presented as mean ± SD (*n* = 3).

Type of MPs	Drug Loading (%) ± SD	Entrapment Efficiency (%) ± SD
PLGA 50:50-based MPs	6.93 ± 1.94	46.71 ± 2.31
PLGA 75:25-based MPs	7.91 ± 2.08	51.63 ± 4.67

## Data Availability

The data supporting the findings of this study are available from the corresponding author upon reasonable request.

## Supplementary Materials

The following supporting information can be downloaded at the following page: https://www.mdpi.com/article/10.3390/pharmaceutics17081066/s1, Figure S1. Experimental profiles of BS release rate (µg/day) from different ocular insert formulations over (A) the first 4 h and (B) the subsequent days.

## Abbreviations

The following abbreviations are used in this manuscript:

**BS**	**Bromfenac sodium**
**DCM**	Dichloromethane
**FDA**	Food and Drug Administration
**MPs**	Microparticles
**PBS**	Phosphate-buffered saline
**PLGA**	Poly(lactic-co-glycolic acid)
**PVA**	Poly(vinyl alcohol)
**SEM**	Scanning electron microscopy
**SMD**	Surface mean diameter
**VMD**	Volume mean diameter
**W/O**	Water-in-oil
**W/O/W**	Water-in-oil-in-water
