# Supplementary material for: Dual-Phase Ocular Insert with Bromfenac-Loaded PLGA MPs in a PVA Matrix for Sustained Postoperative Anti-Inflammatory Delivery
Source: Pharmaceutics. 2025 Aug 17;17(8):1066. doi: 10.3390/pharmaceutics17081066 (PMC12388992; doi:10.3390/pharmaceutics17081066)
Supplement: Supplementary file 1 [file pharmaceutics-17-01066-s001.zip › pharmaceutics-3794800-supplementary.pdf]

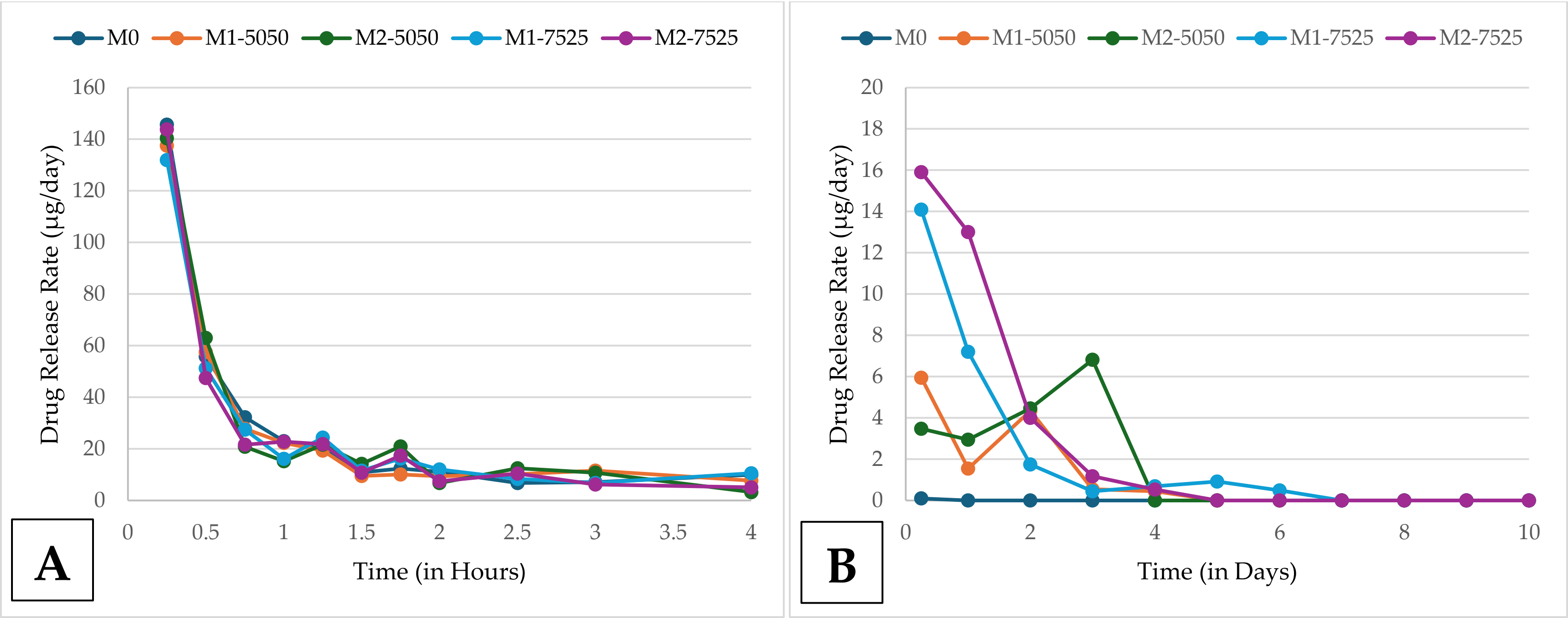

Figure S1. Experimental profiles of BS release rate (µg/day) from different ocular insert formulations over (A) the first 4 hours and (B) the subsequent days.
